# Buddhist-like opposite diminishing and non-judging during ketamine infusion are associated with antidepressant response: an open-label personalized-dosing study

**DOI:** 10.3389/fphar.2022.916641

**Published:** 2022-07-25

**Authors:** Kurt Stocker, Matthias Hartmann, Steffen Reissmann, Andreas Kist, Matthias E. Liechti

**Affiliations:** ^1^ Psychopharmacology Research, Division of Clinical Pharmacology and Toxicology, Department of Clinical Research, University Hospital Basel and University of Basel, Basel, Switzerland; ^2^ Chair of Cognitive Science, Department of Humanities, Social and Political Sciences, ETH Swiss Federal Institute of Technology Zurich, Zurich, Switzerland; ^3^ Department of Psychology, University of Zurich, Zurich, Switzerland; ^4^ Faculty of Psychology, UniDistance Suisse, Brig, Switzerland; ^5^ Medical Office for Anesthesiology Zelenka and Colleagues, Villingen-Schwenningen, Germany

**Keywords:** ketamine, opposite-diminishing, non-judging, depression, Buddhism

## Abstract

**Background:** Cognition that is not dominated by thinking in terms of opposites (opposite diminishing) or by making judgments (non-judging) can be found both in Buddhist/mindfulness contexts and in mental states that are fostered by dissociative psychedelics (*N*-methyl-D-aspartate antagonists) such as ketamine. Especially for the Buddhist/mindfulness case, both opposite diminishing and non-judging have been proposed to relate to mental well-being. Whether ketamine-occasioned opposite diminishing and/or non-judging relate to increased mental well-being in the form of antidepressant response is unknown, and was investigated in the present study.

**Methods:** In this open-label outpatient study, the dose level and frequency for the ketamine infusions were adjusted individually in close consultation with the patients suffering from depression with the overall goal to maximize antidepressant benefits—a novel dose regimen that we term *personalized antidepressant dosing*. In general, treatment started with an initial series of ketamine infusions with a dosage of 0.5 mg/kg body weight and was then adjusted (usually increased). A possible relationship between ketamine-induced antidepressant benefits and retrospectively reported peri-infusion experiences of opposite diminishing and non-judging was assessed based on a total of 45 ketamine-infusion treatment sessions from 11 different patients suffering from depression. Opposite diminishing and non-judging were measured with the two items from the Altered States of Consciousness Inventory (ASCI) that measure these concepts. Depression was measured with the Beck Depression Inventory (BDI-II).

**Results:** Peri-infusion experiences of both opposite diminishing and non-judging were associated with antidepressant responses confirming our hypothesis. Furthermore, opposite diminishing and non-judging were closely related to one another while relating to antidepressant response in distinguishable ways.

**Conclusion:** Future controlled randomized trials with dissociative and other psychedelics and with a larger number of participants are needed to establish the possible link of psychedelically induced opposite diminishing and non-judging with an antidepressant response more firmly.

## Introduction

Descriptions of mental realms where one’s cognition is not dominated by thinking in terms of opposites (opposite diminishing) and where one’s cognition is not dominated by making judgments (non-judging) can be found both in relation to Buddhist meditation and its modern secularized mindfulness forms and in relation to mental states that are occasioned by dissociative psychedelics (*N*-methyl-D-aspartate, NMDA, antagonists) such as ketamine. Additionally, opposite diminishing and non-judging have also been (mostly theoretically) proposed to be associated with increased mental well-being, especially in the Buddhist/mindfulness-meditation case (Kabat-Zinn, 2003; Kornfield, 2008). Although in Buddhist/mindfulness-meditation opposite diminishing, non-judging, and mental well-being are usually characterized to be able to occur in close relation to one another (e.g., Kornfield, 2008, pp. 376–381), it is not known for dissociative psychedelics whether these three characteristics can also occur in relation to one another. This study sets out to investigate these issues with regard to the NMDA receptor antagonist ketamine. This study is to our knowledge the first investigating whether a) the occurrence of opposite diminishing, non-judging, and well-being (measured here as a decrease in depression) can occur in interrelated ways within the ketamine experience, and b) if they do, whether the occurrence of opposite diminishing and/or non-judging can predict antidepressant response in ketamine-treated individuals suffering from depression. In the remainder of this introduction, we first lay out the relevant background information that is needed to understand our experimental hypotheses, before then presenting the experimental hypotheses and how we investigated them.

While the term “mindfulness” is defined differently in Buddhist and modern research-community contexts (Dahl et al., 2015; Brandmeyer et al., 2019), in the research community, it “often refers to a self-regulated attentional stance oriented toward the present-moment experience that is characterized by curiosity, openness, and acceptance” (Dahl et al., 2015, p. 516), which includes “a non-evaluative awareness of one’s thoughts, emotions, and other experiences in the moment” (Lebois et al., 2015, p. 506). Thus, mindfulness involves an accepting, non-evaluative—often also termed a “non-judging” (e.g., Kabat-Zinn, 2003, p. 150)—stance toward one’s current mental life or one’s current life in general.

The non-judging aspect in relation to being mindful is well known and is also covered in most mindfulness questionnaires (Buchheld et al., 2001; Baer et al., 2004, 2006; Bishop et al., 2004; Walach et al., 2006; Feldman et al., 2007; Cardaciotto et al., 2008; Chadwick et al., 2008). However, Buddhist and mindfulness scholars sometimes describe non-judgment to be related to a mental realm where one seems to be no longer thinking about things in terms of opposites. This relationship has not been investigated by the modern mindfulness research community. For exemplification, consider the following passage of Jon Kabat Zinn the founder of the mindfulness-based-stress-reduction program:

So, when we speak of mindfulness as being non-judgmental awareness, it doesn’t mean that there won’t be judgements. It means that you will be aware of how judgmental we actually are, and then “not judge” the judging, and when we relate to it in that kind of a way, then we begin to see that our judging is very often “black and white.” It’s either this or that, good or bad, like—dislike, want—don’t want. And we get imprisoned by that kind of view … being non-judgmental … means that we will cultivate discernment. This is the capacity to see what’s actually unfolding, but not to judge it, but to recognize it, and to understand it, in relationship to our experience … In over days, weeks, months, and years, we can begin to actually find a way to navigate through are judging, in such a way that it no longer dominates our lives in quite the same way, and we recognize when it comes up, that it’s actually in some sense “toxic,” and the more we challenge it, and the more we rest in discernment and in pure awareness, the more we can live life authentically in the present moment, without getting caught by our own “habits of mind”—“unhealthy” if you will, “habits of mind” (Kabat-Zinn (n.d.) in Spiritual Mind, 2022, 0:48).

In this passage, Kabat-Zinn clearly associates being judgmental with thinking in opposites, as he says that judging “very often” involves thinking about things in terms of “black and white.” This also implies that being non-judgmental relates to thinking less (or perhaps at times not at all) in terms of opposites (implies that non-judging relates to opposite diminishing). Kabat-Zinn’s description is also in line with how many non-judging questions are phrased in mindfulness questionnaires. To give one example, four out of the 11 questions that make up the factor “non-judging of experience” in the five facets mindfulness questionnaire are phrased in terms of opposite pairs—e.g., “I make judgments about whether my thoughts are good or bad” (Baer et al., 2006, p. 35). It is important to note, however, that while it is true that opposite diminishing (thinking less in terms of opposites) is often associated with non-judging by mindfulness scholars and in mindfulness research, this usually only happens *implicitly* (by often using opposite examples when describing non-judging, but by not making the opposite topic explicit). To our knowledge, *opposite diminishing as such* is *not* much of a topic in modern mindfulness research, and thus opposite diminishing in itself does also not feature as an explicitly worked out theme in any of the currently used mindfulness questionnaires (eight questionnaires) known to us (Buchheld et al., 2001; Brown and Ryan, 2003; Baer et al., 2004, 2006; Bishop et al., 2004; Walach et al., 2006; Feldman et al., 2007; Cardaciotto et al., 2008; Chadwick et al., 2008).

Unlike in mindfulness psychometrics, the Buddhist scholar Jack Kornfield—a meditation teacher and Buddhist psychologist, who trained as a Buddhist monk and also holds a Ph.D. in clinical psychology—does make opposite diminishing a most central part of a mental realm that can be fostered by Buddhist meditation practice:

BUDDHIST PSYCHOLOGY … shows us the paradox of the universe, within and beyond the opposites. It teaches us to be *in* the world but not *of* the world. This realization is called the middle way … The middle way describes the middle ground between attachment and aversion, between being and non-being, between form and emptiness, between free will and determinism. The more we delve into the middle way the more deeply we come to rest between the play of opposites … To discover the middle way, he [Ajahn Chah, Kornfield’s then meditation master] went on, “Try to be mindful, and let things take their natural course” … The Perfect Wisdom Text describes it [the middle way] as “realization of suchness beyond attainment of good or bad” … Here is a … principle of Buddhist psychology: …The middle way is found between all opposites. Rest in the middle and find well-being wherever you are (2008, pp. 367–369; caps and italics his).

Thus, Kornfield characterizes the aspired Buddhist mental realm (often termed the middle way) as a mental realm in which one does not think of things in terms of opposites, but rather of a mental realm that is “between” or “beyond … opposites” such as “good or bad.” Furthermore, he associates such a mental condition in which thinking about things in terms of opposites diminishes (a state in which we “we come to rest between the play of opposites”) with, one the hand, being non-judgmental (as such a state can be discovered by “let [ting] things take their natural course”), and, on the other hand, also with mental well-being. Indeed, he associates opposite diminishing and mental well-being rather explicitly, when he states above that the “middle way is found between all opposites. Rest in the middle and find well-being wherever you are.”

**FIGURE 1:**
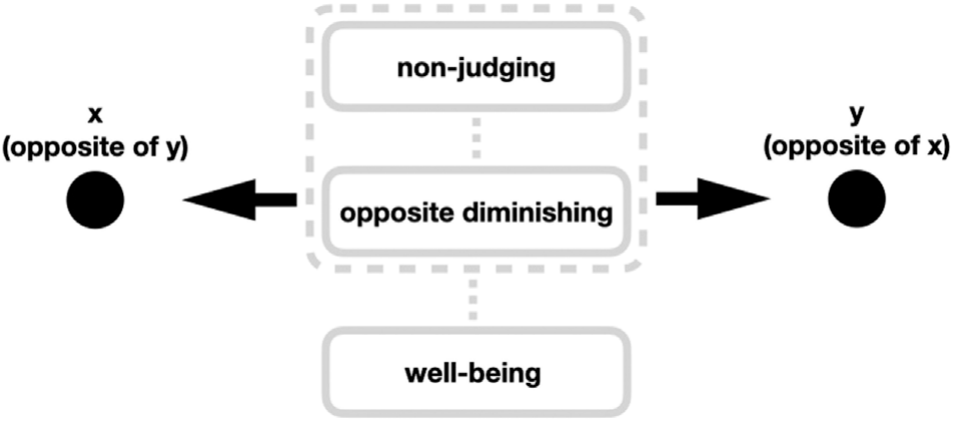
Opposite diminishing is proposed to be a mental realm where one is neither mentally drawn to thinking in terms of one opposite pole of pair of opposites (such as “this is good”, “I like this,” etc.) nor is mentally drawn to thinking in terms of the other opposite pole of the same pair of opposites (such as “this is bad”, “I don’t like this,” etc.). Rather it is a mental realm in which one is located exterior (“between”, “beyond”) to either side of the opposite poles. Such a mental state is proposed to be associated with non-judging, and both of these mental conditions (opposite diminishing and non-judging) are proposed to be associated with mental well-being.

The overall theoretical view that emerges from the observations/descriptions above is that a mental realm that is characterized by opposite diminishing should also be associated with non-judging as well as with mental well-being. Our proposed theoretical model for this view is schematically depicted in Figure 1.

Above Kornfield (and Kabat-Zinn, but more implicitly so) has described opposite diminishing, non-judging, and increased mental well-being as mental aspects that can be achieved through meditation. Interestingly, some first observations suggest that these three mental aspects can also be evoked and investigated by dissociative psychedelics.

The main receptor interaction of dissociative psychedelics such as ketamine and nitrous oxide involves NMDA receptor antagonism (Harrison and Simmonds, 1985; Kalmoe et al., 2020; Krediet et al., 2020). While the group of compounds including ketamine and nitrous oxide is often termed “dissociative anesthetics,” this group is increasingly also (including this paper) alternatively termed “dissociative psychedelics” (cf. Block et al., 1990; Sanz et al., 2018; Krediet et al., 2020)—in order to highlight that the acute experiences of these compounds often do not only include dissociative aspects (such as an out-of-body experience), but also psychedelic aspects other than dissociation such as mystical-type feelings of oneness, visions, and clarity of thought (Studerus et al., 2010). Additionally, these compounds might also be able to occasion opposite diminishing, non-judging, and increased mental well-being.

First, a link between dissociative psychedelics and an acute experience of opposite diminishing has already been described in 1882 by one of the founding fathers of modern psychology, William James. Of course in James’ days, terms like “dissociative anesthetics” or “dissociative psychedelics” were not born yet to refer to such experiences; rather he referred to the nitrous-oxide-occasioned experience as the “the anaesthetic revelation” (James, 1902, p. 389), taking over the term from Blood (1874). He described his self-experiment with nitrous oxide as follows.

Whatever the idea of representation occurred to the mind was seized by the same logical forceps, and served to illustrate the same truth; and that truth was that every opposition, among whatsoever things, vanishes in a higher unity in which it is based; that all contradictions, so-called, are of a common kind; that unbroken continuity is of the essence of being … It is impossible to convey an idea of the torrential character of the identification of opposites as it streams through the mind in this experience. I have sheet after sheet of phrases dictated or written during the intoxication, which to the sober reader seem meaningless drivel, but which at the moment of transcribing were fused in the fire of infinite rationality. God and devil, good and evil, life and death, I and thou, sober and drunk, matter and form, black and white, quality and quantity, shiver of ecstasy and shudder of horror, vomiting and swallowing, inspiration and expiration, fate and reason, great and small, extent and intent, joke and earnest, tragic and comic, and fifty other contrasts figure in these pages in the same monotonous way. The mind saw how each term belonged to its contrast through a knife-edge moment of transition which it effected, and which, perennial and eternal, was the nunc stans of life (1882, pp. 206–207).

At least in this self-experiment case study, the NMDA receptor antagonist nitrous oxide evoked an opposite diminishing of a very strong kind—not only did opposites seem to “vanish,” they also “vanish [ed] in a higher unity” of “unbroken continuity.” James’ initiative to undertake a nitrous-oxide self-experiment was inspired by the reports of Benjamin Blood (1874) about his decade-long nitrous-oxide self-experiments whose subjective experiences can—according to the writer Lachman (2003)—also be interpreted as opposite diminishing:

Blood's anesthetic revelation … is rife with contradictory statements that in any normal discourse would cancel each other out. In Blood's vision, however, they act as a kind of propositional ladder, reaching to a higher, encompassing, all-inclusive reconciliation (2003, p. 17).

Other than these self-experiments of Blood (1874) and James (1882), we are not aware of much other literature that also investigated whether NMDA receptor antagonists such as ketamine or nitrous oxide would occasion the subjective experience of opposite diminishing. However, some more evidence for this is found for ketamine. In relation to ketamine (in healthy participants), a subscale termed “experience of unity”—from a new factor analysis (Studerus et al., 2010) of the Five-Dimensional Altered States of Consciousness Rating Scale (5D-ASC) (Dittrich, 1998; Dittrich et al., 2006, 2010)—showed somewhat higher (not statistically compared) response for ketamine than for the 5-hydroxytryptamine-2A (5-HT_2A_) receptor agonist (classic-psychedelic) psilocybin and the serotonin/dopamine/oxytocin releaser (entactogen/empathogen) 3,4-methylenedioxymethamphetamine (MDMA). The subscale *experience of unity* contains the item “Opposites and contradictions seemed to dissolve”^1^ and therefore asks quite clearly about the occurrence of opposite diminishing. However, since the other items of the subscale *experience of unity* are not about opposite diminishing perception, the relation of ketamine to opposite diminishing as such cannot be discerned from the findings of this study.

Second, the NMDA receptor antagonist ketamine can induce a post-acute experience of *non-judging/acceptance* as illustrated by this qualitative report of an individual suffering from depression. Treated with ketamine therapy, she reported the effects thereof in the following way:

I felt like I was able to deal with the issues in a self-nurturing way. Just that I accepted that all of the stuff that happened, happened and it’s not the end of the world, don’t need to hate myself over things that I haven’t done right (Sumner et al., 2021, p. 954).

This open-minded “I accepted that all of the stuff that happened, happened” clearly seems to resemble Buddhist or mindfulness characterizations of non-judging/acceptance as described above. However, the quoted example was not analyzed in terms of non-judging by the investigators, but in relation to the more abstract notion of “change in their [patients suffering from depression receiving ketamine treatment] feelings around problems in life” (p. 954). Other articles known to us also mention enhanced non-judging/acceptance capacities in relation to ketamine-assisted psychotherapy only in passing (Dore et al., 2019, p. 192; Halstead et al., 2021, p. 320).

Third, there is clear evidence that ketamine can increase well-being. Ketamine produces a rapid antidepressant response in individuals suffering from depression in many studies (e.g., Zarate et al., 2006; Murrough et al., 2013), and the same has been shown for nitrous oxide in a first proof-of-concept study (Nagele et al., 2015; Kalmoe et al., 2020).

While opposite diminishing, non-judging, and mental well-being are usually characterized as being interrelated to one another in the Buddhist/mindfulness case (cf. above), it is not known whether these three characteristics can also occur in interrelated ways with dissociative psychedelics such as ketamine and nitrous oxide. When it comes to dissociative psychedelics and opposite diminishing/non-judging/well-being all we have at hand are some isolated observations/findings for each of these three characteristics as they have been illustrated above. The present study thus sets out to address the question whether the three aspects of opposite diminishing, non-judging, and mental well-being all occur in the ketamine experience, and if they do, how they relate to one another.

The two main hypotheses of the current study are 1) both a ketamine-peri-infusion experience of opposite diminishing as well as a peri-infusion experience of non-judging are associated with increased well-being—measured as an antidepressant response (e.g., Wersebe et al., 2018)—and 2) the experiences of opposite diminishing and non-judging relate to antidepressant benefits in comparable/related, yet still distinct ways.

Hypothesis 1 is based on a Buddhist/mindfulness analogy. Kornfield for instance writes of the “joyful experience of moving … out of duality” (2008, p. 369). And associating Buddhist non-judging with well-being he writes: “It’s our task to learn from the world as it is. For the awakening of the heart, conditions are always good enough” (p. 369). Furthermore, Brown et al. (2015) have found an association between an increase in mindfulness non-judging and a decrease in depressive symptoms and anxiety.

Hypothesis 2 is also based on a Buddhist/mindfulness analogy. As shown above, Buddhist or mindfulness scholars like Kornfield and Kabat-Zinn as well as mindfulness psychometrics suggest (either explicitly or implicitly) that judging often seems to involve thinking in terms of one of two poles of a given opposite pair (e.g., “this is good” vs. “this is bad”). Thus, it is suggested that thinking in terms of opposites and judging—as well as their counterparts opposite diminishing and non-judging—are closely related to one another. In analogy, we suspect that such a close interrelation of opposite diminishing and non-judging also occurs in the ketamine experience because there is evidence of overlap between ketamine-induced and meditation-induced altered-states-of-consciousness phenomenology in domains other than opposite-diminishing/non-judging; for instance, both are known to be involved in dissolving the sense of self (e.g., Studerus et al., 2010; Millière et al., 2018). However, while we expect opposite diminishing and non-judging to closely relate to one another (when one occurs, the other is often likely to occur as well), and while we expect that they both relate to antidepressant response, they should still relate to antidepressant response in somewhat distinguishable ways, since, after all, opposite diminishing and non-judging are two semantically different concepts.

Given what has been said above, opposite diminishing can be characterized as cognition that is not dominated by thinking in terms of opposites. It is a mental realm in which one is located exterior (“between”, “beyond”) to either side of the opposite poles (cf. Figure 1). As such, opposite-diminishing is more of a *perceptual* concept—while in opposite-diminishing one perceives things without applying an opposite-thinking frame to them, the concept *says nothing about one’s attitude* toward whatever is perceived in opposite-diminishing ways. In contrast, also given what has been said above, non-judging can be characterized as cognition that is not dominated by making judgments (which also often involves refraining from applying an opposite-thinking frame on the cognition in question), but non-judging can additionally also be characterized as *accepting* whatever it is that one perceives in non-judging terms. As such, non-judging is also an *attitudinal* concept; although in non-judging (as in opposite-diminishing) one perceives things without applying an opposite-thinking frame on the cognition in question, the concept also *reflects one’s attitude* toward whatever is perceived in non-judging ways—this attitude being an accepting stance toward the cognition is in question.

We addressed the questions of our hypotheses in an open-label, prospective, and non-interventional study. To an existing ketamine treatment regimen of an ambulatory anesthesiological day clinic for patients suffering from depression, we added a new enhanced scale to measure altered states of consciousness (ASCs)—the Altered State of Consciousness Inventory (ASCI) (Reissmann et al., 2022)—and the Beck Depression Inventory II (BDI-II) (Beck et al., 2009). Regarding the dosing regimen, the dose level and frequency for the ketamine infusion were adjusted individually in close consultation with the patient with the overall goal to maximize antidepressant benefit—a novel dose regimen that we term *personalized antidepressant dosing* [for more general aspects on personalized dosing with psychedelics see Liechti and Holze (2021)]. Regarding the measurement of opposite diminishing and non-judging, answers to the two questionnaire items of the ASCI for these concepts were analyzed to see if they relate to the BDI-II scores in the predicted ways. These two ASCI items were “Opposites and contradictions seemed to dissolve” for opposite diminishing, and “I was able to accept my life as it is, without the urge to evaluate it” for non-judging.^2^

The ASCI is a partially new questionnaire that consists of two parts. First, it consists of a validated 11-subscales factor analysis (Studerus et al., 2010) of the 5-Dimensional Altered States of Consciousness Rating Scale (5D-ASC) (Dittrich, 1998; Dittrich et al., 2006, 2010): the 5D-ASC/11. Second, in addition to the 5D-ASC/11, the ASCI contains also new self-generated items whose factor structure has not been investigated yet. However, it is important to highlight that in this given study the factor structure of the ASCI plays no role. We simply selected the two individual items from the ASCI that most closely matched opposite diminishing and non-judging, irrespective of their factor or potential factor structure. If our initial exploration of this topic confirms our hypotheses, then it might be worthwhile to develop more items for both opposite diminishing and non-judging so that they can then also be analyzed on the factor level. For possible development of a non-judging ASC factor in the future, ASC psychometrics could draw inspiration from mindfulness psychometrics, where non-judging and/or acceptance has already been developed into a factor in various ways (Baer et al., 2004, 2006; Cardaciotto et al., 2008).

While the ASCI was administered retrospectively—i.e., shortly after the infusion—the questionnaire still asks about the subjective experiences during the infusion (“Please rate the extent to which the following statements […] apply to your experience during the ketamine infusion”). Asking retrospectively—shortly after the altered state of consciousness (ASC)—about the experiences during the ASC is a standard procedure that applies to most ASC questionnaires.

## Methods

### Participants

Patients suffering from depression who underwent ketamine-infusion therapy at the anesthesiological day clinic Dres. Gugath, Kist, and Schmitz-Buchholz (later Zelenka and colleagues) in Villingen-Schwenningen (Germany), were asked if they would be willing to participate in the current study. The decision of the patients to additionally participate in this study or not did not influence the medical treatment. All participants provided written informed consent after the study procedures were fully explained. The inclusion criteria for the study were 1) a minimum age of 18 years, 2) suffering from depression, and 3) meeting the health requirements for ketamine administration. These health requirements were determined independently of the study, when suitability for ketamine treatment was medically assessed, and mainly included as exclusion criteria acute suicidality, any disease involving the need to avoid excessive increases in blood pressure and/or heart rate, as well as substance use disorders. The present study was authorized by the ethics committee of the Landesärztekammer Baden-Württemberg (Germany). Patients had the possibility to withdraw from the study at any time without giving reasons and without any influence on their medical treatment. The administering physicians were trained anesthesiologists, and consistent medical care was ensured.

Eleven patients (3 females, 8 males) suffering from depression with recurrent major depressive episodes and no resounding therapeutic success before they started the ketamine treatment were included in the study. Their mean age was 48.6 years (ranging from 22 to 70 years; see Table 1 for a summary of demographic information). These patients were diagnosed with depression on average 17.6 years before the start of the ketamine treatment (ranging from 3 to 49; one missing information). Ten out of the 11 patients received at least two previous treatments other than ketamine, whereby only two of those reported reduced depressive symptoms due to these previous treatments.

**TABLE 1:** Mean BDI-II and opposite-dissolving and non-judging values for all patients.

Patient	Age	Gender	NSessions	Dose^a^ (mg/kg)	BDI-II Pre	BDI-II Post	Opposite-dissolving	Non-Judging
				**M [Min, Max, Opt.]**	**M [Min, Max]**	**M [Min, Max]**	**M [Min, Max]**	**M [Min, Max]**
1	39	m	8	2.0 [1.2, 3.1, 3.1]	26.3 [18, 38]	16.0 [7, 23]	3.3 [2, 5]	7.5 [2, 22]
2	62	m	11	0.6 [0.5, 0.9, 0.8]	19.1 [11, 31]	15.4 [4, 37]	14.7 [0, 69]	25.9 [0, 97]
3	69	m	1	0.5 [0.5, 0.5, 0.5]	20.0 [20, 20]	18.0 [18, 18]	6.0 [6, 6]	15.0 [15, 15]
4	31	m	6	0.7 [0.5, 0.8, 0.8]	18.3 [9, 23]	8.0 [1, 16]	38.2 [11, 55]	50.3 [0, 84]
5	27	f	3	0.7 [0.5, 1.0, 1.0]	41.7 [33, 48]	39.7 [32, 46]	3.0 [0, 9]	32.7 [0, 98]
6	22	m	5	n.a.	17.6 [13, 26]	3.0 [0, 9]	92.6 [81, 100]	61.6 [0, 81]
7	65	m	2	0.5 [0.5, 0.6, 0.6]	34.0 [31, 37]	21.0 [13, 29]	31.5 [9, 54]	21.5 [7, 36]
8	30	m	3	0.5 [0.5, 0.5, 0.5]	34.7 [24, 45]	16.0 [7, 23]	41.0 [39, 44]	64.7 [13, 95]
9	54	m	1	0.7 [0.7, 0.7, 0.7]	23.0 [23, 23]	8.0 [8, 8]	63.0 [63, 63]	82.0 [82, 82]
10	70	f	2	n.a.	15.5 [15, 16]	9.0 [8, 10]	35.5 [33, 38]	43.5 [9, 78]
11	66	f	3	n.a.	42.0 [38, 44]	23.3 [16, 36]	0.0 [0, 0]	31.0 [4, 68]

The theoretical score range for the BDI-II, is 0–63 and for the opposite-dissolving/non-judging 0–100. N_Sessions_ is the number of sessions per patient, or respectively the number of data points for each patient that was analyzed. Opt. = optimal dose for each patient (dose used after initial adjustment). Except for one patient, the optimal dose corresponded to the highest dose applied (Max.). The n.a. values are due to missing weight information of three patients.

a The exact dose value for each session, in case that this is not already visible in Table 1, is: Patient 1: 1.2, 1.2, 1.4, 1.7, 2.0, 2.3, 2.5, 3.1. Patient 2: 0.5, 0.5, 0.5, 0.6. 0.5. 0.5, 0.5, 0.5, 0.8, 0.9, 0.9. Patient 4: 0.5, 0.6, 0.6, 0.7, 0.7, 0.8. Patient 5: 0.5, 0.7, 1.0. Patient 8: 0.5, 0.5, 0.6.

Furthermore, some of the patients had other psychiatric diagnoses in addition to suffering from a depressive disorder (e.g., anxiety disorder, post-traumatic stress disorder), but for all patients, the main reason to undergo ketamine treatment was their suffering from depression. Nine out of the 11 patients (one missing information) underwent psychotherapeutic treatment, and seven (one missing information) received medical treatment during the time of the ketamine treatment. Motivated by the lack of therapeutic response to previous pharmacological and psychotherapeutic treatments, patients reached out to the day clinic on their own behalf and were not referred by a psychiatrist or other health professional to the ketamine therapy.

### Personalized antidepressant dosing

For dosing, a personalized regimen was applied. Specifically, the dose level and frequency for the ketamine infusion were adjusted individually in close consultation with the patient with the overall goal to maximize antidepressant benefits and tolerability of the infusion (personalized antidepressant dosing). Treatment started with an initial ketamine infusion (Ketamine Inresa) with a dose of 0.5 mg/kg body weight and a runtime of 40 min to evaluate the patient’s initial response to the treatment—this dosage is an internationally used standard that has been proven to be safe and has often shown antidepressant response (Berman et al., 2000; Zarate et al., 2006; Murrough et al., 2013; Xu et al., 2016; Niciu et al., 2018; Włodarczyk et al., 2021). After these initial (up to three) infusions of 0.5 mg/kg, each patient’s individual response in terms of tolerability and change in depressive symptomatology determined the dose level and frequency of the subsequent infusions. The main criterion for determining the optimal personalized dose was the patients’ subjective feedback on whether they felt that the infusion was sufficient to have had an antidepressant effect. This criterion usually resulted in increasing the initial doses.

Generally, doses were adjusted in one of two basic ways in the dosing regimen of this clinic: 1) in most cases doses were adjusted for the next session based on the patient’s feedback from the previous session, or, 2) less often, doses were also adjusted during the 40-min sessions without altering the runtime.

*Dose adaptions for the next session based on the patient’s feedback from the previous session*. This dose-adaption approach involved talking to the patient shortly after an infusion, and if he/she felt that the acute effect was not sufficient during the infusion, then over the next sessions the dosage was successively increased until a result was achieved that was perceived by the patient as both pleasant and effective in terms of antidepression. Once this result was achieved, then the dose was usually maintained for any further treatments.

*Dose adaptions during the sessions*. This dose-adaption approach involved adjusting the dose during the 40-min ketamine infusion and was used less frequently than adjusting the dosage before the next session. This approach more often involved patients experienced with ketamine infusions who came back regularly for treatment for a longer period of time. At least every 10 min during the infusion (cf. below), the treating physician asked the patient if he/she is doing well, and if he/she thinks that the dose is sufficient. If the impression of the patient was that the antidepressant response was not sufficient, then the dose could also be increased during the infusion. In very rare instances, the dose was also *decreased* during a ketamine infusion. In these rare cases, this was mainly due to either a psychological effect that is too negative (e.g., frightening hallucinations) or as a physical effect that is considered too negative (e.g., hypertension).

### Physiological monitoring

During the ketamine infusion, the patient’s vital signs were monitored by continuous electrocardiogram (ECG) monitoring, pulse oximetry, and blood pressure measurement, complemented with regular checks (at least every 10 min) by the treating physician and/or medical staff of the day clinic. After the infusion, the patient remained in the treatment room or in the waiting area of the day clinic as a precaution. The length of this stay was based on the patient’s tolerance of the infusion and on his/her physical and mental response to the ketamine.

### Questionnaires

In each session, patients completed the BDI-II^3^ before the ketamine infusion (as well as additional questionnaires that were not part of the present study). No later than 40–60 min after the end of the infusion, all mind-altering effects of the ketamine were expected to have subsided (Włodarczyk et al., 2021). Within this post-infusion time window and after inspection by the experienced professional, the ASCI (Reissmann et al., under review) was administered, followed by the second administration of the BDI-II. In the context of this study, only the opposite-diminishing and non-judging items of the ASCI were analyzed (cf. Introduction).

### Analysis

Data from a total of 49 ketamine-treatment sessions were collected. Data from four of these sessions were not included in the analysis because the BDI-II scores prior to the sessions were below the cut-off value of 9 for minimal depression (Wintjen and Petermann, 2010). Thus, a possible relationship between ketamine-induced antidepressant benefits and opposite diminishing and/or non-judging was assessed based on a total of 45 ketamine treatment sessions from 11 different patients. To account for the nested data structure (45 sessions from 11 patients), a linear mixed-effect model was computed with the patient as a random intercept effect. Since there were only one-to-three values for most of the patients, the data was not sufficiently informative to also add a random slope per participant (singular fit issue). Two separate models were computed with either opposite diminishing or non-judging as fixed effect predictors. The dependent variable was the improvement in the BDI-II score after the treatment in % to the pre-treatment BDI-II assessment (baseline). Thus, positive values indicate reduced BDI-II scores, and we consequently expected positive estimates of the fixed effects. The mixed-effect models were computed using the lme4 package in R (Bates et al., 2014). Unstandardized estimates are reported, and *p*-values were calculated using Satterthwaite degrees of freedom. We also report the explained variance (*R* ^*2*^). These values correspond to marginal pseudo-*R* ^*2*^ (i.e., the explained variance based on the fixed effects in the mixed models), as derived from the jtools-package following the procedure described by Nakagawa and Schielzeth (2013).

## Results

When averaged across the 45 sessions, BDI-II scores before ketamine infusion were 24.8 (*SD* = 10.1), and 15.2 (*SD* = 11.0) after the infusion. Thus, ketamine infusion led to an average decrease in BDI-II scores of 9.6 (38.7%). Averaged scores for opposite diminishing were 27.0 (*SD* = 31.9) and 34.8 (*SD* = 34.9) for non-judging. Mean values per patient are summarized in Table 1. There was a significant correlation between opposite diminishing and non-judging, *r*_Spearman_ = 0.61, *p* < 0.001.

**FIGURE 2:**
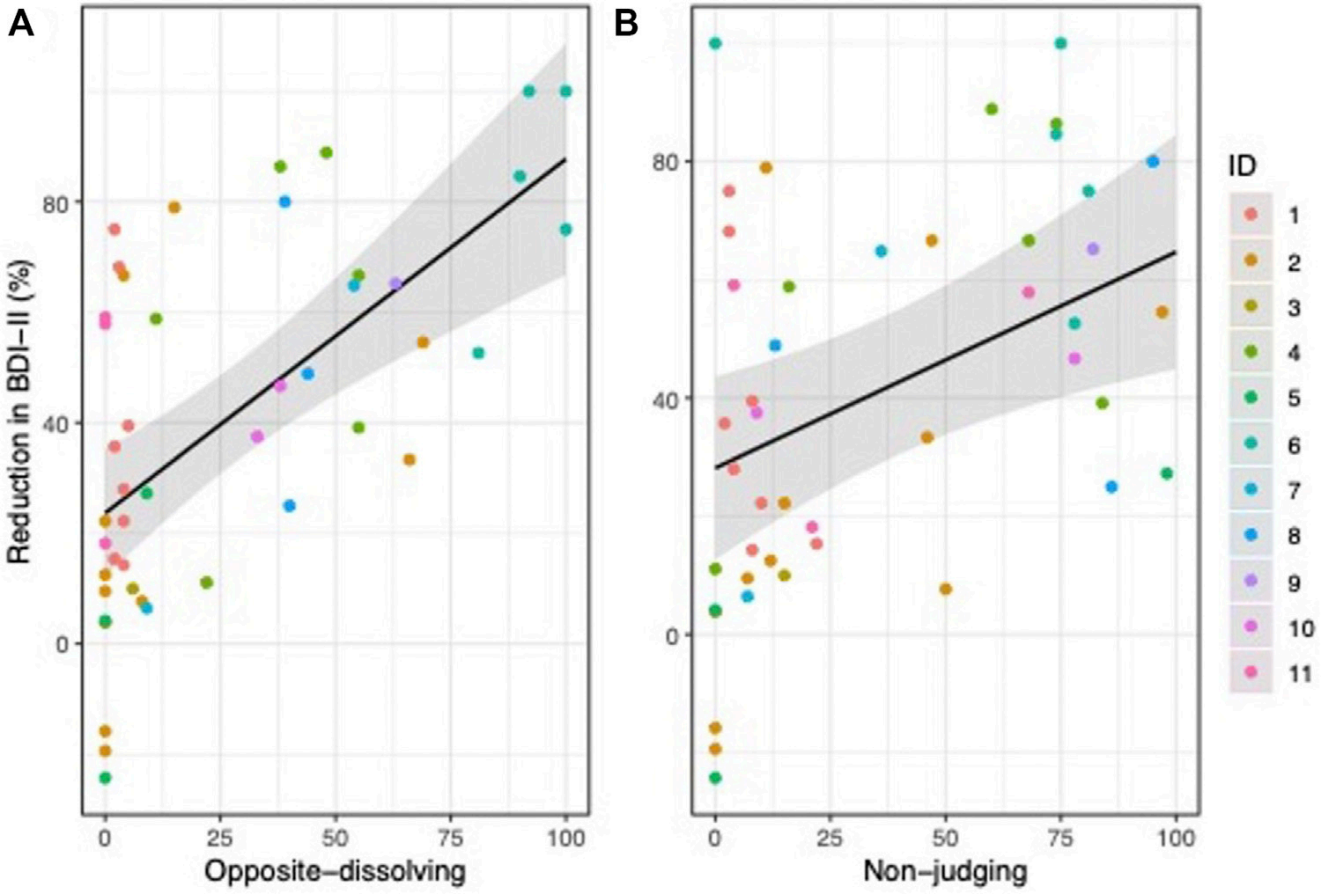
Positive values indicate a reduction in BDI-II score (in % to baseline) following ketamine infusion. Reduction in BDI-II was associated with opposite-dissolving **(A)** and non-judging **(B)**.

Linear mixed-effects analyses revealed significant depression-reduction effects both for opposite diminishing, estimate = 0.64, *SEM* = 0.13, *t* = 4.89, *p* < 0.001, *R*2_marginal_ = 39.1%, and for non-judging, estimate = 0.37, *SEM* = 0.12, *t* = 2.94, *p* = 0.005, *R*2_marginal_ = 15.8%, as illustrated in Figure 2.

As shown in Figure 2, there were some sessions in which patients did not report to experience opposite-diminishing (*n* = 11) or non-judging (*n* = 7) mental realms. In order to show that the results reported above were not biased by these zero values, we repeated the analysis without these datapoints. The results confirmed significant associations with depression-reduction effects for both opposite diminishing, estimate = 0.50, *SEM* = 0.13, *t* = 3.99, *p* < 0.001, *R*2_marginal_ = 32.5%, and non-judging, estimate = 0.31, *SEM* = 0.12, *t* = 2.53, *p* = 0.020, *R* ^*2*^_marginal_ = 15.1%.

When the model with opposite diminishing as a predictor was compared to a model that includes both opposite diminishing and non-judging as predictors, the explained variance in depression-reduction effects did not change (from 39.1% to 39.3% (ΔR = 0.2, χ^2^ (1) = 0.77, *p* = 0.380)). Thus, non-judging did not explain additional variance when opposite diminishing was already used as a predictor. In contrast, when the model with non-judging as a predictor was compared to a model that included both opposite diminishing and non-judging as predictors, the explained variance in the change in BDI-II increased markedly from 15.8% to 39.3% (ΔR^2^ = 23.5, χ^2^ (1) = 10.22, *p* = 0.001).

*Accounting for the distressing experience*. In order to account for possible distressing ketamine-induced ASCs, we created an additional variable that we termed “distressing experience.” As mentioned in the Introduction section, the ASCI also contains all items of the eleven validated subscales from the 5D/ASC-11 (Studerus et al., 2010). Two of these eleven subscales cover distressing ASC experiences: the subscales *anxiety* (6 items) and *impaired control and cognition* (7 items). *Anxiety* contains items like “I was afraid that the state I was in would last forever” or “I felt threatened.” *Impaired control and cognition* contains items like “I felt isolated from everything and everyone” or “I had the feeling that I no longer had a will of my own.” Distressing experience had a mean of 19.1 (*SD* = 17.8). Distressing experience was neither associated with antidepressant effects (spearman rho = -0.03, *p* = 0.843) nor with non-judging (*r*_Spearman_ = 0.10, *p* = 0.509). However, distressing experience was positively associated with opposite-diminishing (*r*_Spearman_ = 0.30, *p* = 0.047). When distressing experience was entered as control variable into the linear mixed effect models, there was still a significant positive association between antidepressant effects and opposite diminishing, estimate = 0.72, *SEM* = 0.12, *t* = 6.08, *p* < 0.001, R2_marginal_ = 46.8%, as well as for non-judging, estimate = 0.34, *SEM* = 0.13, *t* = 2.71, *p* = 0.010, R2_marginal_ = 15.3%. From this additional analysis, we can conclude that distressing ASCs did not play a critical role in the outcome of this study. It should also be noted that distressing psychedelic experiences are not necessarily negative experiences per se (see Discussion).

## Discussion

Our results showed (supporting hypothesis 1) that both the experience of opposite diminishing as well as a mental state of non-judging during the ketamine infusion was significantly associated with reductions in depression scores in individuals suffering from depression. Our finding also showed (supporting hypothesis 2) that opposite-dissolving and non-judging were associated with one another, but that they still did not predict antidepressant response in the same way, especially since non-judging did not explain further variance in a reduction in depression scores when opposite diminishing was already a predictor. Overall, our findings thus give some first empirical support that opposite diminishing and non-judging increase mental well-being in the ketamine experience—as Buddhist/mindfulness scholars propose it for the Buddhist/mindful experience (see Introduction). Why do opposite-diminishing and non-judging seem closely related to one another? To our knowledge, this question has not been addressed much in modern research. However, according to Buddhist or mindfulness scholars like Kornfield and Kabat-Zinn (cf. Introduction), judging often seems to involve thinking in terms of one of two poles of a given opposite pair (e.g., “this is good,” “this is bad”). So, if judging and thinking in terms of opposites might be so closely related to one another, then one could speculate that if thinking in opposites starts to decrease in the Buddhist/mindfulness or the ketamine experience, then judging should correspondingly also decrease. This is supported by our finding that non-judging does not explain further variance in a reduction in depression scores when opposite diminishing is already a predictor. This suggests that the antidepressant effect of non-judging is already covered by opposite diminishing, whereas non-judging alone does not necessarily cover the antidepressant effect of opposite diminishing. Future research is needed to better understand these interactive processes.

Like the NMDA receptor antagonists ketamine and nitrous oxide (Zarate et al., 2006; Murrough et al., 2013; Nagele et al., 2015), 5-HT_2A_ receptor agonists (classic or serotonergic psychedelics) have also shown rapid antidepressant responses in patients with depression (Carhart-Harris et al., 2016; Griffiths et al., 2016). Furthermore, several studies documented increased non-judging (measured with mindfulness questionnaires) after administration of the serotonergic psychedelics ayahuasca and 5-methoxy-*N*,*N*-dimethyltryptamine (Soler et al., 2016, 2018; Sampedro et al., 2017; Uthaug et al., 2018, 2019, 2020; van Oorsouw et al., 2021) (5-MeO-DMT)—see also Watts et al. (2017) for a comparable result in relation to serotonergic psychedelic psilocybin. However, unlike in the current study, non-judging in these studies was not measured in relation to the acute psychedelic phase, but rather for the post-acute phase (“after-glow,” for example, up to 24 h after substance intake) and/or as a follow-up (up to 2 months after substance intake). While the results in these studies are somewhat mixed overall, in most of these studies the classic psychedelics have shown significant increases in non-judging both for the post-acute phase and for the follow-up (including the 2-months follow-up). The finding of our study suggests that it might also be worthwhile for future studies with classic psychedelics to not only measure non-judging for time periods after substance intake, but also for the acute phase. Conversely, the post-substance findings of these classic-psychedelic studies suggest that non-judging should also be investigated for the post-acute phase and as a follow-up for dissociative psychedelics, which is something that has not been done in the current study nor in any other dissociative-psychedelic study that we are aware of. Also, since we found a strong correlation between opposite diminishing and anti-depression in the current study, future studies on classic psychedelics and non-judging could consider broadening their conceptual scope to not only investigate the clinical potential of psychedelically-fostered non-judging (and other mindfulness aspects), but also the clinical potential of psychedelically-fostered opposite diminishing. Opposite diminishing may be a mediator of the therapeutic effects of psychedelics. Additionally, therapeutic responses to psychedelics may be consolidated by non-pharmacological interventions enabling opposite diminishing such as meditation practices.

Furthermore, it is also worthy of note that opposite-diminishing was positively associated with distressing experience. At first sight, this might seem in contradiction with the part of our hypothesis that opposite diminishing is associated with well-being. However, although not studied in enough detail yet, there have been some first observations in relation to classic psychedelics (especially in some qualitative studies) that a distressing/confrontational ASC experience might sometimes be a necessary predecessor of a positive ASC experience. In relation to classic-psychedelic (psilocybin) induced ASCs in psychedelic-assisted treatment of cancer-related distress (Swift et al., 2017) for instance write:

The immersive quality of the experience was felt as overwhelming, challenging, or fearful for many … of the participants, particularly in the early stages of the session … The intensity of this initial phase quickly subsided for all participants who experienced it, and consistently led to significant moments of acceptance, surrender, and new understandings (pp. 497–498).

Richards (2015) who has worked in psychedelic-assisted psychotherapy (with classic psychedelics) for decades sums up his experiences with distressing psychedelic experiences as follows:

[Classic-psychedelic substances] can also trigger personal psychological experiences, such as regression to childhood traumas or confrontation with unresolved grief, fear, anger, or guilt. Such experiences … may well have potentially significant value in accelerating psychotherapy and personal growth … Further, especially if one is unprepared and seeks to control or escape from emerging inner experiences, the flow of unique mental adventures facilitated by psychedelic substances can culminate in episodes of panic, paranoia, confusion, and somatic distress (2015, p. 16)

Whether any of these distressing facets of classic-psychedelically evoked ASCs have also been at work in our dissociative-psychedelic study cannot be determined from our data and has to be left to future investigation. However, we may note that in our study a ketamine-occasioned ASC experience that correlated highly with antidepression—opposite-diminishing—also correlated with ASC distressing experience. Thus, it remains at least a possibility that the distressing experience of our patients reflected more of a constructive type of distressing ASC experience (e.g., necessary confrontational predecessor to a positive experience). Clearly, this merits further research.

As mentioned in the Introduction section, to our knowledge, mindfulness psychometrics does not capture opposite diminishing, and does not directly investigate whether the tendency to think in one pole of given opposite pair is reduced in the mindful trait or state of mind. Given that mindfulness non-judging has been shown to be related to a decrease in depressive symptoms and anxiety (Brown et al., 2015), and given that in our study we did not only find a correlation to antidepressant response in relation to non-judging, but also to opposite-diminishing, we think it would be worthwhile for mindfulness research to start to investigate whether it might be advantageous to integrate opposite diminishing into the mindfulness psychometric research canon. After all, according to the Buddhist psychologist Jack Kornfield (cf. Introduction), a mental realm in which “we come to rest between the play of opposites” (2008, p. 368) lies at the very heart of the Buddhist middle way.

## Limitations

The major limitations of this study were the lack of a control group allowing blinding, a small number of participants (*n* = 11, respectively 45 sessions), and an unbalanced male/female ratio (9 males, 3 females). Therefore, future controlled randomized trials with a larger and more balanced number of participants are needed to establish our findings more firmly. This article also shares the common problem with all other next-generation rapid-acting antidepressant studies, which is the lack of a valid assessment tool that captures short-term (within hours) changes in depressive symptomatology.

In addition, while dosage amount was varied in the dosage regimen of the clinic where the study was conducted, the international standard time of 40 min running time was never varied in our study. We are not aware of any depression ketamine study that systematically has varied ketamine’s running time. Thus, this study shares this limitation—not to have explored different running times to perhaps maximize ketamine’s antidepressant potential—with other ketamine studies with individuals suffering from depression.

Another limitation in relation to the dose regimen is that it is likely that an optimal dose—the dose that was usually kept constant after adjusting the dose according to the subjective feedback of the patient in the first initial sessions—has a stronger effect on ASC experiences (such as opposite diminishing and non-judging) and consequently on depression reduction than a suboptimal dose. However, for the present study, there were not enough data to systematically assess the potential effect of optimal dose.

Furthermore, it should also be kept in mind that our study exclusion criteria did not include other psychiatric disorders (e.g., anxiety disorders or post-traumatic stress disorders). Thus, while the current study can provide some first evidence that ketamine-induced opposite diminishing and non-judging are related to antidepressant response, we did not measure whether ketamine-induced opposite diminishing and non-judging might also be related to improvement in anxiety or PTSD symptoms. Potential clinical benefits of ketamine-induced opposite diminishing and non-judging other than improvements of depressive symptoms is a topic that warrants future study.

Finally, it is also important to keep in mind that our analysis is of correlational nature. One cannot conclude that ketamine-induced opposite diminishing and non-judging caused antidepressant effects. Rather, these specific experiences might also be indicators of how well ketamine generally worked on a physiological level (e.g., the strength of NDMA receptor antagonism) in a given session. However, speaking against such a physiological interpretation is our finding that the associations between opposite diminishing/non-judging hold true even when controlling for other ketamine-induced ASC experiences (distressing ASC effects). This suggests that there is at least a specific relationship between opposite diminishing/non-judging and antidepressant effects.

## Conclusion and outlook

Both descriptions of Buddhist and mindfulness scholars and the results of our present ketamine study suggest that mental states including opposite diminishing may be linked to increased mental well-being. Further studies are needed to clarify the mediating role of opposite diminishing in the benefits of psychedelic-assisted therapy.

## Data Availability

The raw data supporting the conclusion of this article will be made available by the authors, without undue reservation.

## Footnotes

1 The original item used in Studerus et al. (2010) is in German and reads “Gegensätze und Widersprüche schienen sich aufzulösen” (cf. Dittrich et al., 2006) which the authors—using the English translation in relation to Dittrich et al. (2010)—translate as: “Conflicts and contradictions seemed to dissolve.” However, a more accurate English translation of the German word “Gegensätze” than “conflicts” is clearly “opposites” (for “conflicts” there is the German word “Konflikte”). That is why we have rendered the translation of the item used in German in the above text accordingly

2 In the original German version these two items were, respectively: “Gegensätze und Widersprüche schienen sich aufzulösen” and “Ich konnte mein Leben so akzeptieren, wie es ist, ohne den Drang zu haben, es bewerten zu müssen.”

3 As there is no instrument available that measures changes in depressive symptoms within such a short time interval as they can be induced by ketamine, we changed the acquisition period from 2 weeks to 1 h.
